# Giving Children With Osteogenesis Imperfecta a Voice: Participatory Approach for the Development of the Interactive Assessment and Communication Tool Sisom OI

**DOI:** 10.2196/17947

**Published:** 2020-09-22

**Authors:** Maia Siedlikowski, Frank Rauch, Argerie Tsimicalis

**Affiliations:** 1 Ingram School of Nursing Faculty of Medicine and Health Sciences McGill University Montreal, QC Canada; 2 Shriners Hospitals for Children - Canada Montreal, QC Canada

**Keywords:** child health, symptom assessment, communication, mobile apps, software

## Abstract

**Background:**

Children with osteogenesis imperfecta (OI) experience acute and chronic symptoms that expose them to physical, mental, and social challenges. Empowering these children by involving them in their care can help them to cope with OI. Sisom is an interactive assessment and communication tool designed to help children aged 6-12 years with chronic illnesses express their symptoms. This tool has not yet been adapted to the unique needs of OI.

**Objective:**

The aim of this study was to develop a Sisom OI paper prototype by seeking the perspectives of end users.

**Methods:**

A participatory approach was adopted to develop the prototype overseen by an expert panel of 9 clinicians at a university-affiliated pediatric hospital. Purposive sampling was used to recruit 12 children with OI who were aged 6-12 years. The study was carried out over the course of 3 feedback cycles. Data were deductively interpreted using content analysis techniques.

**Results:**

Overall, 64% (57/89) of the Sisom symptoms were deemed relevant for inclusion in Sisom OI, with 42% (37/89) directly incorporated and 22% (20/89) incorporated with changes. In total, 114 symptoms were used to create the prototype, of which 57 were newly generated. The relevant symptoms addressed children’s thoughts and feelings about hospitalization and their wishes for participation in their own care. The new symptoms addressed fractures, body image, and social isolation related to difficulties with accessibility and intimidation.

**Conclusions:**

Once developed, Sisom OI will offer clinicians an innovative and child-centered approach to capture children’s perspectives on their condition.

## Introduction

In health care, children need to be enabled to make their views known on issues that affect them [1,2]. In particular, those living with chronic illnesses have a thorough understanding of their condition [3,4]. This positions them at the center of their own care. Yet, clinicians are confronted with numerous challenges in eliciting these children’s views [5]. There is a risk that their symptoms will be underdiagnosed and undertreated. Children’s perceptions of their symptoms may be inadequately evoked, their rights for participation poorly applied, and thus their needs often neglected [6-10]. A growing body of evidence suggests that interactive software offers an innovative way to enable children to express themselves [11-14].

Sisom (Norwegian acronym derived from “Si det som det er” meaning “Tell it as it is”) is an award-winning, rigorously tested, interactive, computerized tool that helps children aged 6-12 years with chronic illnesses express their symptoms [14]. It is also considered as a creative system to help clinicians better understand children’s perspectives [12]. It utilizes spoken text, sound, and animations to depict symptoms that are each represented by an animated scene within one of the 5 symptom islands. One first creates an “avatar” and is then prompted by Sisom to indicate the presence and severity of the symptoms displayed by using a 5-point Likert-type scale. Upon completion, Sisom generates a child-friendly Symptom Report that can be shared with family and clinicians. The working of Sisom can be viewed on the demo clip available on the internet. Sisom, which was originally designed for children with cancer, adapted for children with congenital heart disease, and in the process of being adapted for children with diabetes, also has the potential to engage children with other chronic conditions to participate in their own care [15].

Osteogenesis imperfecta (OI) is a chronic condition, which has not yet been studied with respect to children’s perceptions of their symptoms. Moreover, to our knowledge, no interactive computerized tools have yet been designed to attend to the unique needs of this population. OI is the most common of the inherited bone disorders and is usually caused by mutations in collagen type I encoding genes [16,17]. The principal clinical feature of OI is bone fragility that leads to frequent fractures. Since there is no cure for OI, health services focus on rehabilitation as well as pharmacological and surgical interventions to prevent or treat fractures and to maximize mobility [18]. The therapeutic goal is an increase in function and a decrease in fractures. However, pain, fatigue, and varying degrees of physical limitations may hinder participation in daily activities, acceptance by peers, and lead to feelings of fear, otherness, and isolation [13,17,19-21]. Overall, relatively little attention has been directed toward understanding the day-to-day symptoms of children with OI from their own perspective.

Sisom addresses the challenges associated with capturing the child’s perspective on their own health status. In studies in Norway and the United States, children with cancer who used Sisom felt better prepared and expressed twice as many symptoms than their peers during “conventional” consults [12,15,22-24]. When oncologists and registered nurses used the Sisom Symptom Report, they asked a large number of clarifying questions, gave more detailed explanations, and communicated with greater empathy, all within the same period allocated for “conventional” consults [12,22-24]. In studies in Canada and the United States, children have expressed an overwhelming interest in using Sisom in a variety of settings such as at home, school, and clinical environments. These children have also remarked the many benefits of Sisom in helping them express themselves [14,22,25].

Adapting Sisom for children with OI has the potential to generate useful and meaningful data that will serve to establish a more comprehensive and “child-friendly” model of care for this population [13]. The purpose of this study was to develop the Sisom OI paper prototype. A participatory approach was used to seek the perspectives of end users, particularly children and clinicians, to inform the development of Sisom OI.

## Methods

### Design

Following institutional review board approval (A06-B29-17B), this descriptive study was conducted at a university-affiliated, nonprofit, pediatric, orthopedic hospital in Montreal, Quebec, specialized in OI care.

### Participants and Recruitment

Recruitment took place between August 2017 and December 2017. Purposive sampling was used to allow for maximum variation in age, self-identified gender, and type of OI for children. A sample size of 10-15 children and 5-10 clinicians was proposed [26,27]. Previous Sisom studies with similar designs as this study have included between 5-12 participants and have successfully established the validity and usability of the tool [14,15,22]. The clinicians were approached by an email sent by a nonauthoritative colleague, not affiliated with the study, and invited to participate. The children with OI were recruited by reaching out to clinicians, who assisted by identifying, screening, and approaching families to determine if they were interested in hearing more about the study. One member of the study team was responsible for providing a verbal and written explanation of the study to those interested in obtaining written informed consent or assent.

### Data Collection and Procedure

This study was carried out over the course of 3 feedback cycles with 2-6 semistructured, face-to-face, individual child interviews per cycle. The parent(s) or legal guardian(s) were given a choice to be present during the interview. The lead author conducted the audio-recorded interviews. The child was invited to use Sisom, which was installed on a laptop, with the lead author. Throughout the interview, children were prompted to answer questions related to the content in Sisom and to consider whether it reflected their own symptoms. Potential drawings that emerged were collected, described, and will be shared in the future with Sisom OI developers. The length of the interview depended on the interest of the child and varied from 20 to 60 minutes in length. Field notes were recorded during and immediately after each interview.

Following each child feedback cycle, the lead author summarized the children’s input on Sisom symptoms, vignettes, and avatars, and hosted an expert panel meeting wherein the synthesized data generated from each child feedback cycle were relayed back to the clinicians for input. Moreover, any discrepancies, similarities, or ambiguities in the children’s responses were discussed. These meetings were carried out with all clinicians in person as one group at the study site and were facilitated by the lead author and another member of the study team. Clinicians were also given the opportunity to view Sisom themselves and were prompted to consider changes that they thought were relevant to OI. Field notes were recorded during and immediately after each meeting. Multiple data sources were collected and they included the self-reported sociodemographic questionnaires for clinicians and children, the Sisom checklists for clinicians and children (Multimedia Appendix 1), transcribed audio recordings from child interviews, written summaries of the audio recordings from expert panel meetings, and field notes from child interviews and expert panel meetings, which included detailed descriptions of nonverbal data, other observations, impressions, and any drawings generated.

### Data Analysis

In the following analyses, a symptom is defined as any question asked by Sisom. Self-report sociodemographic questionnaire data were descriptively analyzed to characterize our samples. Data analysis occurred concurrently with data collection [28,29].

### Expert Panel Meetings

During the first meeting, clinicians viewed Sisom and used their clinical expertise to categorize symptoms according to the following predetermined mutually exclusive categories: (1) Relevant, (2) Irrelevant, (3) To modify, (4) To add, and (5) Unsure. Any symptoms initially coded as “Unsure” were subsequently coded into one of the other categories by following the procedures of data triangulation. During the second expert panel meeting, data collected from the second cycle of the child interviews were shared and critically examined. This contributed to establishing the focus of the third cycle of child interviews. During the third expert panel meeting, data collected from the third cycle of the child interviews were shared and a comprehensive list of Sisom OI symptoms was established. The study team then reviewed this list for feasibility and the final Sisom OI paper prototype was created. During the fourth expert panel meeting, the final Sisom OI paper prototype was shared with the clinicians.

### Child Interviews

These data were analyzed according to island, deductively, by using content analysis techniques. Guided by the following framework, data pertaining to symptoms, vignettes, or avatars were coded into 5 categories for each island: (1) Relevant, (2) Irrelevant, (3) To modify, (4) To add, and (5) Unsure. The children’s rationales for coding the content were highlighted by matching their quotes to the corresponding symptoms, vignettes, or avatars. Drawings were matched to corresponding symptoms and used to showcase children’s suggestions of vignettes for future development. Following the conclusion of each cycle, the child interviews for that cycle were compiled and synthesized in preparation for the next expert panel meeting.

### Integration of Expert Panel Meetings and Child Interviews

Integration consisted of an iterative process of data reduction of transcripts, summaries, and field notes; data display in the form of lists, tables, and figures; conclusion drawing from recurrent patterns; and verification by drawing contrasts [30]. Constant comparative methods were applied within each cycle. This involved a comparison of elements present in one data source with those in another to determine similarities [28]. In this fashion, commonalities were identified among data sources. The content that clinicians, children, and the study team all agreed on was the symptoms that were considered significant and ranked the highest in terms of priority for software development. The content was then integrated and tabulated to create the Sisom OI paper prototype (Multimedia Appendix 1). An audit trail composed primarily of methodological and analytical documentation was kept, which permitted the reproducibility and the transferability of the process [31].

## Results

### Sample Characteristics

In total, 12 children participated in this study (Table 1). No child withdrew from the study. Altogether, 9 clinicians participated in this study (Table 2). The clinician participation rate was 100% (9/9). No clinician withdrew from the study. The clinicians recruited included 4 nurses, 1 physiotherapist, 1 occupational therapist, 1 social worker, 1 special education teacher, and 1 child life specialist.

**Table 1:** Demographic characteristics of the children (n=12).

Characteristics		Values
Age (years), range 6-12, mean (SD)		9 (2)
Number of family members living at home, range 2-6, mean (SD)		4 (1)
**Gender, n (%)**		
	Male	7 (58)
	Female	5 (42)
**Nationality, n (%)**		
	Provincial (Quebec)	5 (42)
	National (Canada)	3 (25)
	International	4 (33)
**Language(s) spoken at home, n (%)**		
	English	4 (33)
	French	4 (33)
	Bilingual (English and French)	1 (8)
	Bilingual (English and other)	3 (25)
**Current fracture, n (%)**		
	Yes	5 (42)
	No	7 (58)
**Use of mobility device(s), n (%)**		
	Wheelchair	2 (17)
	Wheelchair and walker	4 (33)
	None	6 (50)
**Use of computer or tablet at school, n (%)**		
	Yes	9 (75)
	No	3 (25)
**Use of computer or tablet at home, n (%)**		
	Yes	12 (100)
	No	0 (0)
**Use of own mobile phone, n (%)**		
	Yes	5 (42)
	No	7 (58)

**Table 2:** Demographic characteristics of the clinicians (n=9).

Characteristics		Values
Full-time employment, n (%)		9 (100)
**Experience**		
	Number of years in profession, range 7-35, mean (SD)	20 (11)
	Cumulative number of years in profession	176
	Number of years in OI^a^ care for children, range 3-30, mean (SD)	15 (11)
	Cumulative number of years in OI care for children	136

^a^OI: osteogenesis imperfecta.

### Expert Panel Meeting Findings

In the first meeting, clinicians revealed that they were keen to implement Sisom OI into their practice. The clinicians also assessed the 89 Sisom symptoms for relevance. The first cycle of child feedback was shared with clinicians who highlighted the importance of having Sisom OI adopt a more positive lens. Syntax changes were suggested. Then, a new list of OI-specific symptoms was created. Based on their clinical expertise, clinicians identified several islands that would benefit from further child input: “My Body,” “At The Hospital,” and “About Managing Things.” During the second meeting, clinicians agreed on the need to capture the diverse experiences of pain in OI within the “My Body” island. The clinicians suggested that the “At the Hospital” subisland be divided into several core areas based on the health care trajectory of a child who arrives with a fracture. In the third meeting, minor changes were made to the “The Bathroom” subisland’s symptoms to reflect the extra help required for mobilization. The clinicians suggested the creation of a new subisland in “About Managing Things” called “Getting Around.” This was in order to capture the importance of accessibility for children with OI and the challenges associated with engaging in leisure activities in public spaces. During the fourth meeting, the clinicians reviewed all Sisom OI content. This meeting concluded with a rich discussion about implications for practice. It was acknowledged that factors such as context of completion, level of parent involvement, and level of clinician involvement would all depend on the child.

### Child Interview Findings

**Figure 1:**
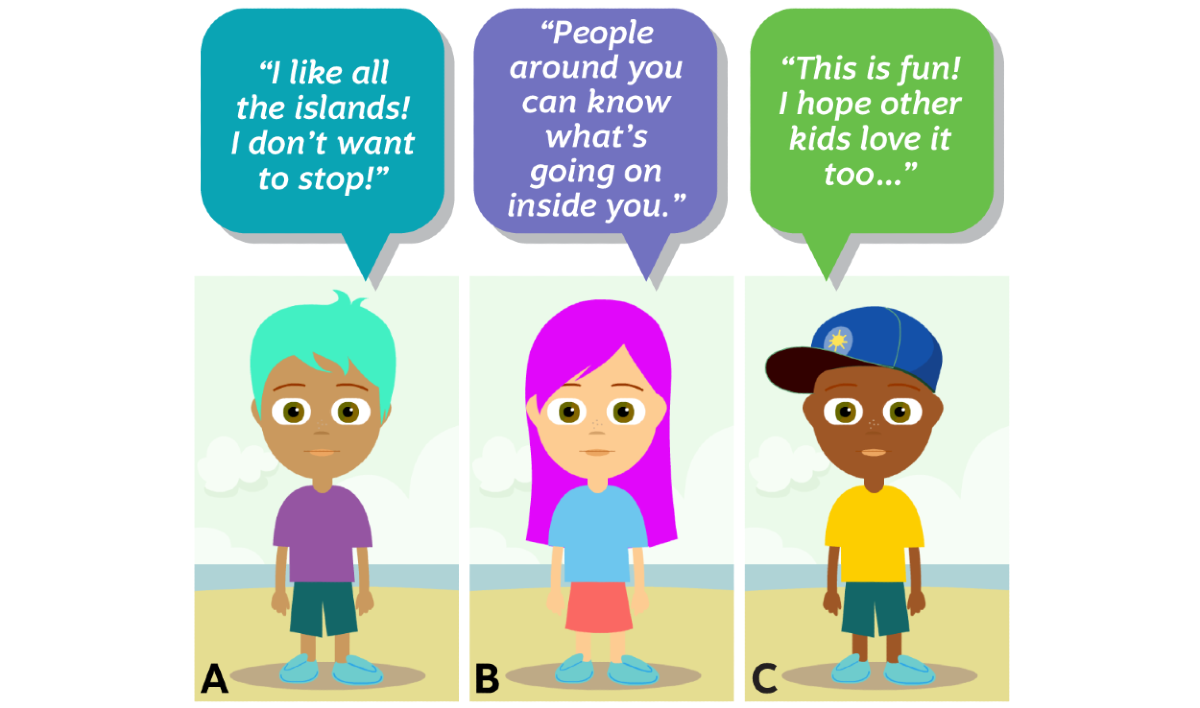
General impressions of Sisom. A. Quote by Participant #6, 6 years old. B. Quote by Participant #4, 8 years old. C. Quote by Participant #5, 10 years old.

These findings are summarized according to Sisom islands. Half of the children (n=6) were accompanied by one of their parents during their interviews. There were 4 mothers and 2 fathers. All children enjoyed using Sisom and completed it with excitement (Figure 1). One-third of the children (n=4) expressed themselves through songs during their Sisom journey. One-third of the children (n=4) chose to check their Sisom Symptom Reports. Finally, some children discussed implementation wishes (Figure 1).

### Avatar

All children expressed a strong desire for the avatar to reflect their identity, which they defined by their physical appearance, inside and out, and by their preferences. Several children explained what helped support their bodies from the inside (eg, surgical rods) and from the outside (eg, mobility devices). To create space for the gender spectrum, “Choose Boy or Girl” was removed. There were 3 main additions: “Choose your current mood,” “Choose what helps you get around,” and “Choose what helps support your body” to celebrate their uniqueness.

Makes you feel like you’re not like everybody else. You can see yourself through a computer technological program!

Participant #4, 8 years old

### About Me

Several children spoke of the importance of creating a space to help others get to know them.

I think there should be one more island about your imagination and what you expect from others. There should also be an area where you can express your feelings about who you are.

Participant #5, 10 years old

In light of this, 3 main additions were made: “Here you can tell about your imagination, your wishes, and your dreams,” “Here you can tell about your family,” and “Here you can tell about your friends” (Table 3). Table 3 illustrates the symptoms from Sisom that were deemed “Relevant” and directly incorporated into Sisom OI, the symptoms that were deemed “To Modify” and incorporated into Sisom OI with changes to syntax, answer options, location, or vignette, and the symptoms that were “To Add,” that is, entirely newly generated symptoms from the child feedback cycles overseen by the expert panel. Two children spontaneously made the same suggestion of creating a space to journal their answers to allow for flexibility through drawn, written, or spoken entries.

**Table 3:** Composition of the Sisom OI islands.

Island			Total number of symptoms		Relevant symptoms, n (%)		Modified symptoms, n (%)		New symptoms, n (%)
**Avatar**									
	Sisom	4		1 (25)		2 (50)		N/A^a^	
	OI^b^ version	8		N/A		N/A		5 (63)	
**About Me**									
	Sisom	0		0 (0)		0 (0)		N/A	
	OI version	3		N/A		N/A		3 (100)	
**At the Hospital**									
	Sisom	18		7 (39)		2 (11)		N/A	
	OI version	19		N/A		N/A		10 (53)	
**My Body**									
	Sisom	28		12 (43)		4 (14)		N/A	
	OI version	28		N/A		N/A		12 (43)	
**About Managing Things**									
	Sisom	21		6 (29)		7 (33)		N/A	
	OI version	27		N/A		N/A		14 (52)	
**Thoughts and Feelings**									
	Sisom	10		7 (70)		3 (30)		N/A	
	OI version	18		N/A		N/A		8 (44)	
**Things One Might Be Afraid of**									
	Sisom	8		4 (50)		2 (25)		N/A	
	OI version	11		N/A		N/A		5 (45)	
**Total**									
	Sisom	89		37 (42)		20 (22)		N/A	
	OI version	114		N/A		N/A		57 (50)	

^a^Not applicable.

^b^OI: osteogenesis imperfecta.

### At the Hospital

Overall, 50% (9/18) of the “At the Hospital” symptoms were retained. However, this whole island was reorganized to fit the experiences of children with OI.

Sometimes I say the hospital and I are friends cause OI kids go to the hospital a lot for check-ups and stuff.

Participant #5, 10 years old

**Figure 2:**
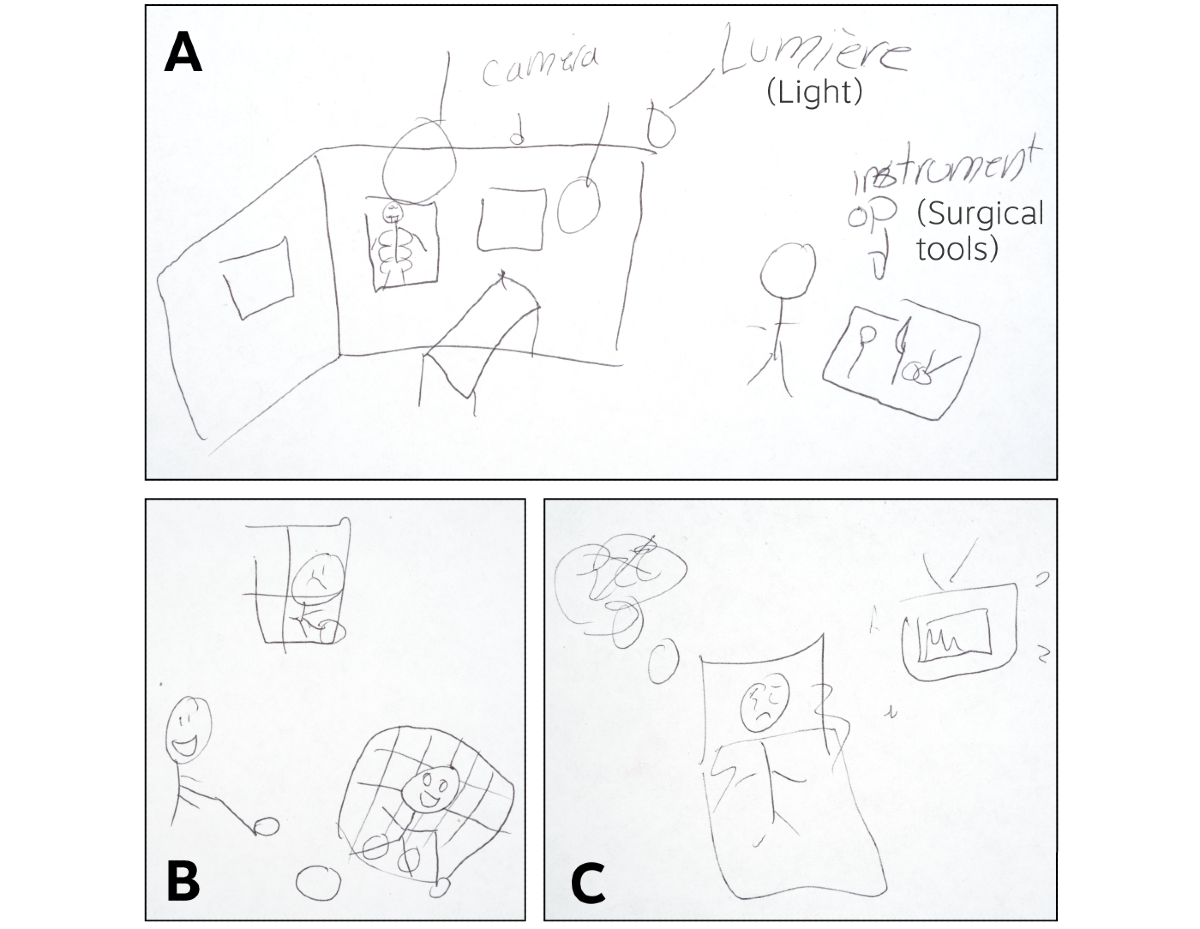
Suggested new symptoms and the corresponding vignettes. A. How is it for you to enter the operating room? Participant #11, 11 years old: “In the room, there are lots of lights. There is a bed and sometimes, you can see the staff looking at their sharp silver instruments and that’s stressful for a child. There are also cameras and radiographic images! Those can be really scary.” B. How do you feel when you are not able to do certain activities? Participant #11, 11 years old: “Show someone in a cast looking out of the window sadly watching their friends play hockey. It affects you to see your friends happy while you have to stay inside doing nothing. That’s it…” C. How is it for you to wake up after a surgery? Participant #11, 11 years old: “Show someone that is not well. Show the machines that beep. See? He’s moving. He’s in pain. Show someone who wants to sleep but can’t.”.

The new subislands included “The Clinic and The Unit,” “In The Operating Room,” “The Cast Room,” and “The Rehabilitation Room.” On this island, children were able to say with certainty about the symptoms that they had never experienced, for instance, “How is it for you to get a feeding tube?” These symptoms that the children had never experienced were removed (Table 4). This table illustrates the symptoms from Sisom that were retained in Sisom OI, that is, deemed “Relevant” or “To Modify” as well as the symptoms that were deemed “Irrelevant” and eliminated by the child feedback cycles overseen by the expert panel. The subisland “About Making Your Own Decisions” was preserved. One child provided vignette suggestions for “How does it feel like when you enter the operating room?”, “How do you feel after you wake up after a surgery?”, and “How do you feel with a cast?” (Figure 2).

**Table 4:** Transferability of Sisom symptoms to the Sisom OI paper prototype.

Island	Total number of symptoms	Irrelevant symptoms, n (%)	Retained symptoms, n (%)
Avatar	4	1 (25)	3 (75)
At the Hospital	18	9 (50)	9 (50)
My Body	28	12 (43)	16 (57)
About Managing Things	21	8 (38)	13 (62)
Thoughts and Feelings	10	0 (0)	10 (100)
Things One Might Be Afraid of	8	2 (25)	6 (75)
Total	89	32 (36)	57 (64)

### My Body

Overall, 57% (16/28) of the “My Body” symptoms were retained. Most revisions and additions reflected the need to capture the children’s use of mobility devices, their accessibility issues, and their constipation issues. Some changes also reflected the perception the children shared about the importance of their bones.

There should be an ‘x-ray’ button!

Participant #7, 9 years old

You could show where you often break so that you could explain that’s where you are the most fragile.

Participant #11, 11 years old

The vignette for the “Pain and Discomfort” subisland was thus modified to allow children to view a skeleton to help them identify the exact bones that caused them discomfort.

### About Managing Things

Overall, 62% (13/21) of the “About Managing Things” symptoms were retained. Here, children attributed the greatest importance to the “School Yard” subisland where the symptoms “Do you ever feel left out?” and “Do others ever bully you?” garnered the most attention.

Ok. There should definitely be an area for how to handle bullies.

Participant #5, 10 years old

**Figure 3:**
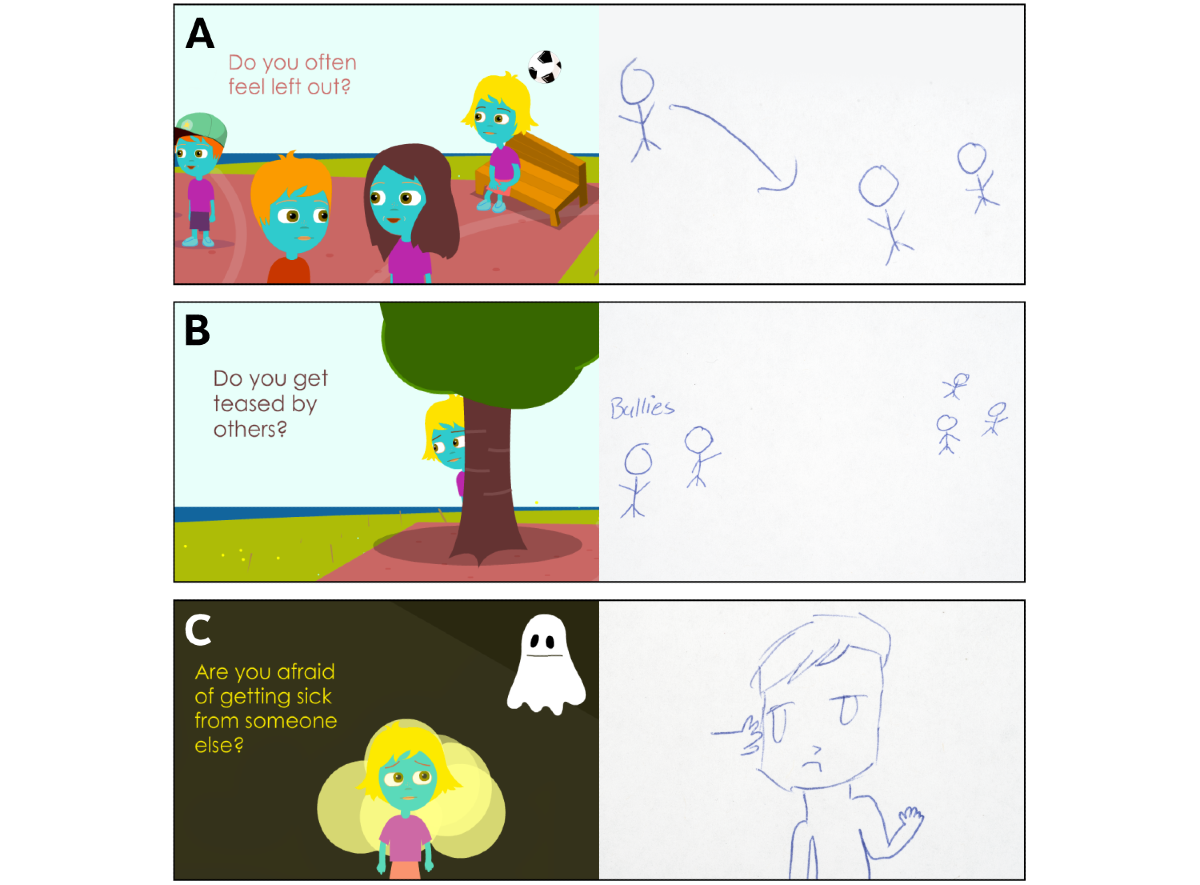
Suggested modifications to symptoms and the corresponding vignettes. A. “Do you ever feel left out?” changed from “Do you often feel left out?” Participant #5, 10 years old: “Instead of showing someone sitting on a bench, show a person walking up to a group of friends and when that person tries to talk to them, they just go somewhere else. That makes you feel left out...” B. “Do others ever bully you?” changed from “Do you get teased by others?” Participant #5, 10 years old: “Instead of someone hiding behind a tree, there should be bullies teasing and pointing.” C. “Are you afraid of getting hurt by someone?” changed from “Are you afraid of getting sick from someone else?” Participant #5, 10 years old: “Show a person sitting down who is being punched".

One child provided vignette modifications for these 2 symptoms (Figure 3). One child stated:

I can tell that people often speak about me and laugh at me (…) No one wants to be with me because of my disease.

Participant #8, 7 years old

Another reflected aloud:

I get teased a lot. I am starting to realize this is not a world where nice people are (…). There won’t always be someone to support you.

This narrative was common among all participants. Children also shared challenges related to their independence, their social lives, as well as the gazes of others:

People give me strange looks at the store and I find that very unpleasant...When I go to the restaurant, people often stare at me. I don’t like that.

Participant #11, 11 years old

Further additions were made “At Home” and “At School” to reflect accessibility issues:

We need a lot of help because of our condition. These questions are very good questions.

Participant #5, 10 years old

### Thoughts and Feelings

Overall, 100% (10/10) of the “Thoughts And Feelings” symptoms were retained. Feelings of otherness expressed by the children were what drove the changes made to this island. Their perceptions of uniqueness were viewed both positively and negatively. In response to “Do you feel different from the other children?”, one child pointed out,

Ya but I think it’s a good thing. I think that because I’m in a wheelchair I can do things other kids can’t do… But being in a wheelchair also has its disadvantages.

Participant #5, 10 years old

Further additions reflected children’s experiences of having to cope with others’ invasive questions about their bodies.

### Things One Might Be Afraid Of

Altogether, 75% (6/8) of the “Things One Might Be Afraid Of” symptoms were retained. The main revisions and additions on this island reflected the unique nature of OI as a health condition. One child provided a symptom and the corresponding vignette modification suggestion for “Are you afraid of getting sick from someone else?” to “Are you afraid of getting hurt by someone else?” (Figure 3). Others explained as follows:

Overall I’m most scared of fractures.

Participant #2, 9 years old

When I get a fracture, sometimes it’s really bad and I can hear that snap! It makes me a bit shivery just thinking about it…

Participant #7, 9 years old

Overall, this was the island that most children visited first and the only island that several children visited more than once.

## Discussion

### Brief Summary of the Findings

Overall, 64% (57/89) of the Sisom symptoms were deemed relevant for inclusion in Sisom OI, with 42% (37/89) directly incorporated and 22% (20/89) incorporated with changes. The relevant symptoms addressed children’s thoughts and feelings about hospitalization and wishes for participation in their own care. In total, 114 symptoms were used to create the prototype among which 57 were newly generated. These new symptoms addressed fractures, body image, and social isolation related to difficulties with accessibility and intimidation. This indicates the need to create Sisom OI to attend to all the specific needs of this population. These new symptoms may also represent the experiences of other children that have similar negative experiences because of societal structures and attitudes premised upon ableism [32].

### Children With Chronic Conditions Share Symptoms

The overwhelming number of symptoms that were transferable from Sisom to Sisom OI show that children, regardless of their specific health condition, want to be involved in decisions that affect them, have similar fears within the health care setting, and experience challenges with integration among peers [8,33]. These findings are supported by the literature in which children with other chronic conditions, life-long illnesses, and learning difficulties share similar illness experiences [3,4,34,35]. Some common themes that emerged from qualitative studies with these populations include an aspiration for “normalcy,” a life of ups and downs, and changes for the whole family [3,34,36,37]. Other common narratives include a desire to be included and informed, develop assertiveness, gain responsibility, live daily life in stride, and participate in social activities [3,4,34,35,38].

### Symptoms as Positive and Negative Experiences

Currently, Sisom assumes that if a symptom is present, it is a source of distress in either a mild, moderate, or severe way. Yet, children explained that the presence of a symptom was not necessarily lived as a disturbance. This finding was also reported in previous Sisom studies in which children emphasized the need for Sisom to use neutral language [14,15,22,25].

In the literature, symptoms experienced by children have been described as “feeling states” lived as a function of context as opposed to isolated measurable sensations assumed to be sources of suffering [37,38]. The children in this study wanted Sisom OI to embrace an outlook in which they could describe their sources of happiness, pride, and support. This discourse is one that is shared by the OI community. By adopting a positive outlook on life, individuals with OI can live their lives to the fullest, despite the many difficult symptoms they experience [39].

### Children as Partners in Research and Design

Our participatory approach builds upon how Sisom was originally created [12,40,41]. The principles we applied reflect those described in the Agile Manifesto under which requirements and solutions evolve through the collaborative effort of the end users [42-50]. Clinicians were touched by the children’s personal stories about themselves and their relationships, insights about their OI, and deep understanding of their strengths and challenges [1,8,51]. Together, end users agreed that children’s experiences of stigma and resulting feelings of otherness must be addressed as early as possible [13]. With the addition of the “About Me” island and the revisions to the “About Managing Things” island, Sisom OI will have the potential to screen for these particular experiences and offer clinicians an opening to address what is of concern to the child.

### Limitations

One important factor in any study is the quality of the target group representation during investigations. In this study, we have involved few participants. One criterion that was used to judge that enough data had been collected to conduct an analysis was data saturation, which was reached at the point where a sense of closure was attained because new data from our multitude of sources yielded redundant information [26-28]. Multiple data sources provided an opportunity to evaluate the extent to which a consistent and coherent picture of the content to include in the Sisom OI paper prototype emerged [28]. In the future, this prototype is to be subjected to further verification, validation, and evaluation. In this study, the views of the primary caregivers of these children were not elicited [34,52]. To what extent disruptions of interviews by primary caregivers had an impact on the children’s participation remains unknown. Our anecdotal reports suggest that those caregivers who were present expressed interest in using Sisom OI to enhance their children’s communication of symptoms. This was a similar finding to those from other Sisom studies [12,14,15,22,24]. Future research will incorporate their views.

### Implications for Clinical Practice

This work demonstrates that it is feasible to involve children and clinicians in the creation of software designed for them. Once fully developed, there is potential that the data generated by Sisom OI be collected centrally in order to track trends in the symptoms of an individual, community, or population. Sisom OI may improve communication between children and their clinicians as encountered in previous Sisom studies and become fully integrated into the practice setting such as in Norway [12,14,15,22,24]. It may also enhance children’s participation in their own care by promoting discussions about what these children deem most important. Empowering children by actively involving them in their care may help them to cope with the difficult physical, mental, and social challenges they face. This, in turn, may ease the transfer of self-management skills, ultimately resulting in a greater quality of life.

### Conclusion

Interactive software such as Sisom offer a solution to assessing the complex symptoms of children with OI and eliciting their perspectives on their health. The successful Sisom tool that addresses the child directly has the potential to change the communication patterns between children and clinicians and could strengthen children’s empowerment [53,54]. Future directions for this work include an inductive analysis of what the children shared when consulted as partners in the development of the Sisom OI paper prototype. It will also be necessary to secure the funds required to create Sisom OI, subject it to further testing, determine incorporation into practice, and evaluate outcomes.

## Appendix

Multimedia Appendix 1 Sisom OI paper prototype.

## Abbreviations

OI: osteogenesis imperfecta

## References

[1] United Nations (1990). Convention on the Rights of the Child.

[2] United Nations (2003). The State of the World's Children.

[3] Alderson P, Sutcliffe K, Curtis K (2006). Children as partners with adults in their medical care. Arch Dis Child.

[4] Sartain S, Clarke C, Heyman R (2000). Hearing the voices of children with chronic illness. J Adv Nurs.

[5] Van Dulmen AM (1998). Children's contributions to pediatric outpatient encounters. Pediatrics.

[6] Söderbäck Maja, Coyne I, Harder M (2011). The importance of including both a child perspective and the child's perspective within health care settings to provide truly child-centred care. J Child Health Care.

[7] Kelsey J, Abelson-Mitchell N (2007). Adolescent communication: perceptions and beliefs. Journal of Children's and Young People's Nursing.

[8] Carnevale FA, Campbell A, Collin-Vézina D, Macdonald ME (2013). Interdisciplinary Studies of Childhood Ethics: Developing a New Field of Inquiry. Child Soc.

[9] Peña Ana L Noreña, Rojas JG (2014). Ethical aspects of children's perceptions of information-giving in care. Nurs Ethics.

[10] Virkki M, Tolonen T, Koskimaa T (2015). Children as decision-makers in health care: An integrative review. Clinical Nursing Studies.

[11] Driessnack M (2005). Children's drawings as facilitators of communication: A meta-analysis. J Pediatr Nurs.

[12] Ruland CM, Starren J, Vatne TM (2008). Participatory design with children in the development of a support system for patient-centered care in pediatric oncology. J Biomed Inform.

[13] Tsimicalis A, Denis-Larocque G, Michalovic A, Lepage C, Williams K, Yao T, Palomo T, Dahan-Oliel N, Le May S, Rauch F (2016). The psychosocial experience of individuals living with osteogenesis imperfecta: A mixed-methods systematic review. Qual Life Res.

[14] Tsimicalis A, Stone PW, Bakken S, Yoon S, Sands S, Porter R, Ruland C (2014). Usability testing of a computerized communication tool in a diverse urban pediatric population. Cancer Nurs.

[15] Arvidsson S, Gilljam B, Nygren J, Ruland CM, Nordby-Bøe Trude, Svedberg P (2016). Redesign and validation of Sisom, an interactive assessment and communication tool for children with cancer. JMIR Mhealth Uhealth.

[16] Bardai G, Moffatt P, Glorieux FH, Rauch F (2016). DNA sequence analysis in 598 individuals with a clinical diagnosis of osteogenesis imperfecta: Diagnostic yield and mutation spectrum. Osteoporos Int.

[17] Dahan-Oliel N, Oliel S, Tsimicalis A, Montpetit K, Rauch F, Dogba MJ (2016). Quality of life in osteogenesis imperfecta: A mixed-methods systematic review. Am J Med Genet A.

[18] Trejo P, Rauch F (2016). Osteogenesis imperfecta in children and adolescents: New developments in diagnosis and treatment. Osteoporos Int.

[19] Shapiro JE, Germain-Lee E L (2012). Osteogenesis imperfecta: Effecting the transition from adolescent to adult medical care. J Musculoskelet Neuronal Interact.

[20] Fegran L, Hall EO, Uhrenfeldt L, Aagaard H, Ludvigsen MS (2014). Adolescents' and young adults' transition experiences when transferring from paediatric to adult care: A qualitative metasynthesis. Int J Nurs Stud.

[21] Nghiem T, Louli J, Treherne SC, Anderson CE, Tsimicalis A, Lalloo C, Stinson J, Thorstad K (2017). Pain experiences of children and adolescents with osteogenesis imperfecta: An integrative review. Clin J Pain.

[22] Tsimicalis A, Le May S, Stinson J, Rennick J, Vachon M, Louli J, Bérubé Sarah, Treherne S, Yoon S, Nordby Bøe Trude, Ruland C (2017). Linguistic validation of an interactive communication tool to help French-speaking children express their cancer symptoms. J Pediatr Oncol Nurs.

[23] Tsimicalis A, Stone PW, Bakken S, Yoon S, Sands S, Porter R, Ruland C (2014). Usability testing of a computerized communication tool in a diverse urban pediatric population. Cancer Nurs.

[24] Baggott C, Baird J, Hinds P, Ruland CM, Miaskowski C (2015). Evaluation of Sisom: A computer-based animated tool to elicit symptoms and psychosocial concerns from children with cancer. Eur J Oncol Nurs.

[25] Tsimicalis A, Rennick J, Le May S, Stinson J, Sarkis B, Séguin K, Siedlikowski M, Choquette A, Louli J (2019). “Tell it as it is”: How Sisom prompts children and parents to discuss their cancer experience. Cancer Reports.

[26] Jakob Nielsen (2000). Nielsen Norman Group World Leaders in Research-Based User Experience. Why You Only Need to Test with 5 Users.

[27] Nielsen JT, Landauer TK (1993). A Mathematical Model of the Finding of Usability Problems.

[28] Polit DC, Beck CT (2017). Nursing Research: Generating and Assessing Evidence for Nursing Practice. Nursing Research: Generating and Assessing Evidence for Nursing Practice.

[29] Thorne S (2000). Data analysis in qualitative research. Evid Based Nurs.

[30] Miles MB, Huberman AM (1994). Qualitative Data Analysis: An Expanded Sourcebook. Qualitative Data Analysis: An Expanded Sourcebook.

[31] Rodgers BL, Cowles KV (1993). The qualitative research audit trail: A complex collection of documentation. Res Nurs Health.

[32] Policy on Ableism and Discrimination Based on Disability. Ontario Human Rights Commission.

[33] King G, Batorowicz B, Rigby P, Pinto M, Thompson L, Goh F (2014). The leisure activity settings and experiences of youth with severe disabilities. Dev Neurorehabil.

[34] Garth B, Aroni R (2003). 'I Value What You have to Say'. Seeking the Perspective of Children with a Disability, Not Just their Parents. Disability & Society.

[35] Livesley J, Long T (2013). Children's experiences as hospital in-patients: Voice, competence and work. Messages for nursing from a critical ethnographic study. Int J Nurs Stud.

[36] Dogba MJ, Bedos C, Durigova M, Montpetit K, Wong T, Glorieux FH, Rauch F (2013). The impact of severe osteogenesis imperfecta on the lives of young patients and their parents: A qualitative analysis. BMC Pediatr.

[37] Woodgate RL, Degner LF, Yanofsky R (2003). A different perspective to approaching cancer symptoms in children. Journal of Pain and Symptom Management.

[38] Wyness M (2009). Adults' involvement in children's participation: Juggling children's places and spaces. Children and Society.

[39] Care 4 Brittle Bones Ambassadors. Care 4 Brittle Bones.

[40] Ruland C (2002). Handheld technology to improve patient care: Evaluating a support system for preference-based care planning at the bedside. J Am Med Inform Assoc.

[41] Ruland CM, White T, Stevens M, Fanciullo G, Khilani SM (2003). Effects of a computerized system to support shared decision making in symptom management of cancer patients: Preliminary results. J Am Med Inform Assoc.

[42] Beck K, Beedle M, Van BA 2001. Manifesto for Agile Software Development.

[43] Steele GC, Khan AI, Kuluski K, McKillop I, Sharpe S, Bierman AS, Lyons RF, Cott C (2016). Improving patient experience and primary care quality for patients with complex chronic disease using the electronic patient-reported outcomes tool: Adopting qualitative methods into a user-centered design approach. JMIR Res Protoc.

[44] Kildea J, Battista J, Cabral B, Hendren L, Herrera D, Hijal T, Joseph A (2019). Design and development of a person-centered patient portal using participatory stakeholder co-design. J Med Internet Res.

[45] Yardley L, Morrison L, Bradbury K, Muller I (2015). The person-based approach to intervention development: Application to digital health-related behavior change interventions. J Med Internet Res.

[46] (1994). The CHAOS Report. The Standish Group.

[47] Bate P, Robert G (2006). Experience-based design: From redesigning the system around the patient to co-designing services with the patient. Qual Saf Health Care.

[48] Boyd H, McKernon S, Mullin B, Old A (2012). Improving healthcare through the use of co-design. N Z Med J.

[49] Christensen T (2017). The evolution of patient-centered care and the meaning of co-design. Institute for Healthcare Improvement.

[50] Chokshi SK, Mann DM (2018). Innovating from within: A process model for user-centered digital development in academic medical centers. JMIR Hum Factors.

[51] Davis JM (1998). Understanding the meanings of children: a reflexive process. Children & Society.

[52] Gannoni AF, Shute RH (2010). Parental and child perspectives on adaptation to childhood chronic illness: A qualitative study. Clin Child Psychol Psychiatry.

[53] McNaughton D, Light J (2013). The iPad and mobile technology revolution: Benefits and challenges for individuals who require augmentative and alternative communication. Augment Altern Commun.

[54] Raaff C, Glazebrook C, Wharrad H (2014). A systematic review of interactive multimedia interventions to promote children's communication with health professionals: Implications for communicating with overweight children. BMC Med Inform Decis Mak.

