# Supplementary material for: Giving Children With Osteogenesis Imperfecta a Voice: Participatory Approach for the Development of the Interactive Assessment and Communication Tool Sisom OI
Source: J Med Internet Res. 2020 Sep 22;22(9):e17947. doi: 10.2196/17947 (PMC7539168; doi:10.2196/17947)
Supplement: Multimedia Appendix 1 [file jmir_v22i9e17947_app1.docx]

**Multimedia Appendix 1 – Sisom OI Paper Prototype**

Symptoms are displayed according to island and labelled as ‘Relevant’ (i.e. Directly incorporated from Sisom into Sisom OI), ‘Modified’ (i.e. Modified from Sisom with changes to syntax, answer options, location, and/or vignette and included in Sisom OI), or ‘New’ (i.e. Entirely newly generated content proposed by child feedback cycles overseen by the expert panel and included in Sisom OI).

| **Sisom OI** | **RELEVANT**  **From Sisom** | **MODIFIED**  **From Sisom** | **PROPOSED**  **NEW ADDITION** |
| --- | --- | --- | --- |
| **AVATAR** | **x** |  |  |
| Choose your skin colour. | **x** |  |  |
| Choose your eye colour. |  |  | **x** |
| Choose your shirt colour. |  | **x** |  |
| Choose what you would like on your head. |  | **x** |  |
| Choose what you would like on your feet. |  |  | **x** |
| Choose your current mood. |  |  | **x** |
| What helps you get around? |  |  | **x** |
| What helps support your body? |  |  | **x** |
| **ABOUT ME** |  |  | **x** |
| Here you can tell about your imagination, your wishes, and your dreams. |  |  | **x** |
| Here you can tell about your family. |  |  | **x** |
| Here you can tell about your friends. |  |  | **x** |
| **AT THE HOSPITAL** | **x** |  |  |
| **THE CLINIC AND THE UNIT** |  | **x** |  |
| How it is for you to check your blood pressure? | **x** |  |  |
| How is it for you to take a blood test? | **x** |  |  |
| How is it for you to get an x-ray? |  |  | **x** |
| How is it for you to get treatment? |  | **x** |  |
| How is it for you to get a needle into the port? | **x** |  |  |
| How is it for you to get an IV? | **x** |  |  |
| How is it for you to remove a band-aid? | **x** |  |  |
| How is it for you to take medications? | **x** |  |  |
| **IN THE OPERATING ROOM** |  |  | **x** |
| How is it for you to prepare for surgery? |  |  | **x** |
| How is it for you to enter the operating room? |  |  | **x** |
| How is it for you to get anesthesia? | **x** |  |  |
| How is it for you to wake up after surgery? |  |  | **x** |
| How is it for you to remove stitches or staples? |  | **x** |  |
| **THE REHABILITATION ROOM** |  |  | **x** |
| How is it for you to practice walking? |  |  | **x** |
| How is it for you to practice exercising? |  |  | **x** |
| How is it for you work out in the pool? |  |  | **x** |
| **THE CAST ROOM** |  |  | **x** |
| How is it for you to have a cast? |  |  | **x** |
| How is it for you to have to stay in the same position? |  |  | **x** |
| How is it for you to not be able to do certain activities? |  |  | **x** |
|  |  |  |  |
| **Sisom OI** | **RELEVANT**  **From Sisom** | **MODIFIED**  **From Sisom** | **PROPOSED**  **NEW ADDITION** |
| **ABOUT MAKING YOUR OWN DECISIONS** | **x** |  |  |
| Are you unsure of what you want? | **x** |  |  |
| Is it difficult to tell others how you feel? | **x** |  |  |
| Would you like to be alone more often? | **x** |  |  |
| Do you miss home? | **x** |  |  |
| Would you like to make your own choices more often? | **x** |  |  |
| Is it difficult to get answers to your questions? | **x** |  |  |
| Do you feel like others do not listen to you? |  |  | **x** |
| Do you feel like others do not explain what they are doing to you? |  |  | **x** |
| **MY BODY** | **x** |  |  |
| **PAIN AND DISCOMFORT** |  | **x** |  |
| Show where you have bruises. | **x** |  |  |
| Show where you have an itch. | **x** |  |  |
| Show where you have pain. | **x** |  |  |
| **THE BATHROOM** | **x** |  |  |
| How is it for you to ask for help to go to the bathroom? |  |  | **x** |
| Do you need help to transfer in the bathroom? |  |  | **x** |
| Do you need help to wipe in the bathroom? |  |  | **x** |
| How is it for you to use public bathrooms? |  |  | **x** |
| How is it to use your other people’s bathrooms? |  |  | **x** |
| Do you hold in your pee or poop? |  | **x** |  |
| Is it difficult to poop? |  |  | **x** |
| Does it hurt to poop? | **x** |  |  |
| **HOW MY BODY WORKS** |  | **x** |  |
| How is it to breathe? |  | **x** |  |
| How is it to walk? |  | **x** |  |
| How is it to run? |  | **x** |  |
| How is it to wheel? |  |  | **x** |
| I think I am slow to move. |  |  | **x** |
| **HOW I LOOK** | **x** |  |  |
| I think I am fat. | **x** |  |  |
| I think I am thin. | **x** |  |  |
| I think I am short. |  |  | **x** |
| I think I have many scars on my body. |  |  | **x** |
| I think I have many broken bones in my body. |  |  | **x** |
| **HOW MY BODY FEELS** | **x** |  |  |
| Feeling sick. | **x** |  |  |
| Feeling hot or sweaty. | **x** |  |  |
| Feeling like throwing up. | **x** |  |  |
| Feeling dizzy. | **x** |  |  |
| Feeling pins and needles in my arms and legs. | **x** |  |  |
| Feeling clumsy. | **x** |  |  |
| Feeling fragile. |  |  | **x** |
| **Sisom OI** | **RELEVANT**  **From Sisom** | **MODIFIED**  **From Sisom** | **PROPOSED**  **NEW ADDITION** |
| **ABOUT MANAGING THINGS** | **x** |  |  |
| **EATING AND DRINKING** | **x** |  |  |
| How is it for you to eat? |  | **x** |  |
| How it is for you to chew your food? |  |  | **x** |
| How is it for you to sit at a table? |  |  | **x** |
| How is it for you to go to the restaurant? |  |  | **x** |
| **AT SCHOOL** | **x** |  |  |
| How is it for you to get around at school? |  |  | **x** |
| How is it for you to have an attendant with you? |  |  | **x** |
| How is it for you to concentrate? | **x** |  |  |
| How is it for you to write? | **x** |  |  |
| How is it for you to complete your work in a short period of time? |  |  | **x** |
| How is it for you to work for a long period of time? | **x** |  |  |
| How is it for you to talk to teachers at school? |  |  | **x** |
| How is gym class for you? |  |  | **x** |
| **SCHOOL YARD** | **x** |  |  |
| How is it for you to keep up with others? |  | **x** |  |
| Is it hard to carry your school bag? | **x** |  |  |
| Do you ever feel left out? |  | **x** |  |
| Do others ever bully you? |  | **x** |  |
| How is it for you to ask others for help? |  |  | **x** |
| Do you easily get tired? |  | **x** |  |
| How is it for you to play with others? |  |  | **x** |
| **AT HOME** | **x** |  |  |
| Is it hard for you to fall asleep? | **x** |  |  |
| Is it hard for you to relax? | **x** |  |  |
| How is it for you to reach things by yourself? |  |  | **x** |
| Do you need help washing? |  | **x** |  |
| Do you need help dressing? |  | **x** |  |
| **GETTING AROUND** |  |  | **x** |
| How is it for you to get around in the streets? |  |  | **x** |
| How is it for you to go to your friends' and family's homes? |  |  | **x** |
| How is it for you to go out? |  |  | **x** |
| **THOUGHTS AND FEELINGS** | **x** |  |  |
| **FAMILY AND FRIENDS** | **x** |  |  |
| Do you believe others are upset because of your OI? |  | **x** |  |
| Do you think that it’s your fault that you have OI? |  | **x** |  |
| Do you feel different from other children? | **x** |  |  |
| Do you miss your family or friends? | **x** |  |  |
| Do you get along with your siblings? |  |  | **x** |
|  |  |  |  |
| **Sisom OI** | **RELEVANT**  **From Sisom** | **MODIFIED**  **From Sisom** | **PROPOSED**  **NEW ADDITION** |
| **FEELINGS** | **x** |  |  |
| Tired of talking and nagging. | **x** |  |  |
| Feel lonely. | **x** |  |  |
| Get angry easily. | **x** |  |  |
| Get sad easily. | **x** |  |  |
| Feel that nothing is fun. |  | **x** |  |
| Feel bored. |  |  | **x** |
| Wonder what other might think of you. | **x** |  |  |
| Tired of asking for help. |  |  | **x** |
| Tired of others asking your questions. |  |  | **x** |
| Tired of explaining yourself to others. |  |  | **x** |
| Tired of others staring at you. |  |  | **x** |
| Feel proud of myself. |  |  | **x** |
| Feel happy for myself. |  |  | **x** |
| **THINGS ONE MIGHT BE AFRAID OF** | **x** |  |  |
| Afraid of walking into a room full of strangers. |  |  | **x** |
| Afraid of getting hurt by someone else. |  | **x** |  |
| Afraid of getting a fracture. |  |  | **x** |
| Afraid of having surgery. |  |  | **x** |
| Afraid of maybe dying from my OI. |  | **x** |  |
| Afraid that the treatment will make me more sick. | **x** |  |  |
| Afraid but don't know why. | **x** |  |  |
| Afraid of being alone. | **x** |  |  |
| Afraid I won't be able to do what I want because of my OI. |  |  | **x** |
| Afraid of doing certain activities after getting hurt. |  |  | **x** |
| Afraid of nightmares. | **x** |  |  |
